# Mechanical Thrombectomy in Prestroke Disability: Data From the Italian Endovascular Stroke Registry

**DOI:** 10.1161/STROKEAHA.124.048997

**Published:** 2025-03-07

**Authors:** Andrea Naldi, Federico D’Agata, Giovanni Pracucci, Valentina Saia, Roberto Cavallo, Davide Castellano, Fabrizio Sallustio, Ilaria Casetta, Enrico Fainardi, Valerio Da Ros, Ilaria Maestrini, Sergio Lucio Vinci, Paolino La Spina, Nicola Limbucci, Patrizia Nencini, Elvis Lafe, Marco Longoni, Sandra Bracco, Rossana Tassi, Stefano Vallone, Guido Bigliardi, Paolo Cerrato, Lucio Castellan, Massimo Del Sette, Roberto Menozzi, Alessandro Pezzini, Stefano Merolla, Stefano Forlivesi, Sergio Nappini, Nicola Davide Loizzo, Andrea Saletti, Cristiano Azzini, Guido Andrea Lazzarotti, Nicola Giannini, Daniele Giuseppe Romano, Rosa Napoletano, Nicola Burdi, Giovanni Boero, Alessio Comai, Elisa Dall’Ora, Nicola Cavasin, Adriana Critelli, Mauro Plebani, Manuel Cappellari, Domenico Sergio Zimatore, Marco Petruzzellis, Francesco Biraschi, Ettore Nicolini, Antioco Sanna, Tiziana Tassinari, Edoardo Puglielli, Alfonsina Casalena, Ivan Gallesio, Delfina Ferrandi, Pietro Filauri, Simona Sacco, Adriana Paladini, Annalisa Rizzo, Michele Besana, Alessia Giossi, Marco Pavia, Paolo Invernizzi, Pietro Amistà, Monia Russo, Marco Filizzolo, Marina Mannino, Gianluca Galvano, Eleonora Lidia Saracco, Mauro Bergui, Salvatore Mangiafico, Danilo Toni

**Affiliations:** Neurology Unit (A.N., R.C.), San Giovanni Bosco Hospital, Turin, Italy.; Department of Interventional Radiology and Neuroradiology (D.C.), San Giovanni Bosco Hospital, Turin, Italy.; Department of Neuroscience, University of Turin, Italy (F.D.).; Department of NEUROFARBA, Neuroscience Section, University of Florence, Italy (G.P.).; Neurology and Stroke Unit, Santa Corona Hospital, Pietra Ligure, Italy (V.S., T.T.).; Unità di Trattamento Neurovascolare, Ospedale dei Castelli-ASL6, Rome, Italy (F.S.).; Neurology Unit, University Hospital Arcispedale S. Anna, Ferrara, Italy (I.C.).; Dipartimento di Scienze Biomediche, Sperimentali e Cliniche, Neuroradiologia, Università degli Studi di Firenze, Ospedale Universitario Careggi, Firenze, Italy (E.F.).; Dipartimento di Biomedicina e Prevenzione, UOSD Radiologia Interventistica, Rome, Italy (V.D.R.).; Department of Systems Medicine, University of Rome Tor Vergata, Italy (I.M.).; Neuroradiology Unit, Department of Biomedical, Dental and Morphological and Functional Imaging Sciences, University of Messina, Italy (S.L.V.).; UOSD Stroke Unit AOU G. Martino, Messina, Italy (P.L.S.).; Interventional Neurovascular Unit, Careggi University Hospital, Florence, Italy (N.L.).; Stroke Unit, Azienda Ospedaliero Universitaria Careggi, Firenze, Italy (P.N.).; Neuroradiologia Interventistica AUSL Romagna, Cesena, Italy (E.L.).; Neurologia e Stroke Unit Ospedale Bufalini, Cesena, Italy (M.L.).; UOC Neuroradiologia diagnostica e terapeutica AOU Senese, Siena, Italy (S.B.).; UOC Stroke Unit AOU Senese, Siena, Italy (R.T.).; UO Neuroradiologia (S.V.), Ospedale Civile di Baggiovara, AOU Modena, Italy.; Neurologia-Stroke Unit (G. Bigliardi), Ospedale Civile di Baggiovara, AOU Modena, Italy.; AO Città della Salute, Torino, Italy (P.C.).; UO Neuroradiologia (L.C.), IRCCS Ospedale Policlinico San Martino, Genova, Italy.; UO Neurologia (M.D.S.), IRCCS Ospedale Policlinico San Martino, Genova, Italy.; Unità Complessa di Neuroradiologia, Azienda Ospedaliero-Universitaria, Parma, Italy (R.M.).; Department of Medicine and Surgery, University of Parma, Italy (A. Pezzini).; Stroke Care Program, Department of Emergency, Parma University Hospital, Italy (A. Pezzini).; IRCCS Istituto delle Scienze Neurologiche di Bologna, UOSI Neuroradiologia Ospedale Maggiore, Bologna, Italy (S. Merolla).; Department of Neurology and Stroke Center, IRCCS Istituto delle Scienze Neurologiche di Bologna, Maggiore Hospital, Italy (S.F.).; Radiologia e Neuroradiologia diagnostica e interventistica, IRCCS Policlinico San Matteo, Pavia, Italy (S.N.).; UO Neurologia d’Urgenza e Stroke Unit, IRCCS Fondazione Mondino, Pavia, Italy (N.D.L.).; UO Neuroradiologia Dip (A. Saletti), Neuroscienze AZOU Ferrara, Italy.; UO Neurologia Dip (C.A.), Neuroscienze AZOU Ferrara, Italy.; Neuroradiology Unit, University Hospital of Pisa, Italy (G.A.L.).; Neurological Institute, Azienda Ospedaliero Universitaria Pisana, Italy (N.G.).; UOSD Interventistica AOU, Salerno, Italy (D.G.R.).; UOSD Stroke Unit AOU San Giovanni di Dio e Ruggi d’Aragona, Salerno, Italy (R.N.).; UOC Neuroradiologia (N.B.), Ospedale SS Annunziata, Taranto, Italy.; UOC Neurologia (G. Boero), Ospedale SS Annunziata, Taranto, Italy.; Departments of Neuroradiology (A. Comai), Hospital of Bolzano (SABES-ASDAA), Teaching Hospital of Paracelsus Medical University, Bolzano, Italy.; Neurology (E.D.), Hospital of Bolzano (SABES-ASDAA), Teaching Hospital of Paracelsus Medical University, Bolzano, Italy.; UO Neuroradiologia Ospedale dell’Angelo, Mestre, Italy (N.C.).; UO Neurologia Ospedale dell’Angelo, Mestre, Italy (A. Critelli).; Neuroradiology Department, Verona, Italy (M. Plebani).; Stroke Unit, Azienda Ospedaliera Universitaria Integrata, Verona, Italy (M.C.).; UO Neuroradiologia AOU Consorziale Policlinico, Bari, Italy (D.S.Z.).; UOC Neurologia e Stroke Unit “Puca” AOU Consorziale Policlinico, Bari, Italy (M. Petruzzellis).; Department of Human Neurosciences, Interventional Neuroradiology (F.B.), Sapienza University, Rome, Italy.; Department of Human Neurosciences (E.N.), Sapienza University, Rome, Italy.; SC Neuroradiologia Diagnostica e interventistica, Ospedale Santa Corona, Pietra Ligure, Italy (A. Sanna).; ASL Teramo, Italy (E.P., A. Casalena).; S.C. Radiologia e Interventistica AOU “SS. Antonio e Biagio e Cesare Arrigo”, Alessandria, Italy (I.G.).; S.C. Neurologia AOU “SS. Antonio e Biagio e Cesare Arrigo”, Alessandria, Italy (D.F.).; Neuroradiology Unit, Presidio Ospedaliero Santi Filippo e Nicola di Avezzano, Italy (P.F.).; Neuroscience Section, Department of Applied Clinical Sciences and Biotechnology, University of L’Aquila, Italy (S.S.).; Departments of Neuroradiology (A. Paladini), Vito Fazzi Hospital, Lecce, Italy.; Neurology (A.R.), Vito Fazzi Hospital, Lecce, Italy.; U.O Neuroradiologia, Dipartimento di Neuroscienze, Presidio Ospedaliero di Cremona (M. Besana), ASST Cremona, Italy.; UOC Neurologia e Stroke Unit (A.G.), ASST Cremona, Italy.; Fondazione Poliambulanza, Brescia, Italy (M. Pavia).; UO Neurologia, Istituto Ospedaliero Fondazione Poliambulanza, Brescia, Italy (P.I.).; UOC Neuroradiologia (P.A.), Ospedale Santa Maria della Misericordia, Rovigo, Italy.; UOS Stroke Unit (M.R.), Ospedale Santa Maria della Misericordia, Rovigo, Italy.; UO Radiologia (M.F.), A.O.O.R. Villa Sofia-Cervello, Palermo, Italy.; UOC Neurologia con Stroke Unit (M.M.), A.O.O.R. Villa Sofia-Cervello, Palermo, Italy.; UO Neuroradiologia (G.G.), ARNAS Garibaldi, Catania, Italy.; UO Neurologia (E.L.S.), ARNAS Garibaldi, Catania, Italy.; Dipartimento di Neuroscienze, Università di Torino, Italy (M. Bergui).; Interventional Neuroradiology Consultant at IRCCS Neuromed, Pozzilli (IS), and Adjunct Professor of Interventional Neuroradiology at Tor Vergata University, Sapienza University and S. Andrea Hospital, Rome, Italy (S. Mangiafico).; Emergency Department Stroke Unit, Department of Human Neurosciences, Sapienza University of Rome, Italy (D.T.).

**Keywords:** cerebral infarction, intracranial hemorrhages, stroke, thrombectomy, thrombolytic therapy

## Abstract

**BACKGROUND:**

The benefits and safety of mechanical thrombectomy (MT) in patients with prestroke disability, classified as modified Rankin Scale (mRS) score of 3 to 4, and anterior circulation stroke remain uncertain. This study aims to evaluate these factors using data from the Italian Registry of Endovascular Treatment in Acute Stroke.

**METHODS:**

We analyzed data collected between 2015 and 2021, comparing functional outcomes (mRS), symptomatic intracerebral hemorrhage, and recanalization rates (Thrombolysis in Cerebral Infarction) at 90 days post-MT in patients with prestroke mRS score of 3 to 4 versus 0 to 2. A good outcome was defined as no change in the mRS score from baseline. Subgroup analysis was stratified by age.

**RESULTS:**

A total of 11.411 (96%) patients with prestroke mRS score of 0 to 2 and 477 (4%) patients with prestroke mRS score of 3 to 4 were included. Compared with patients with a baseline mRS score 0 to 2, those with mRS score 3 to 4 were older (82 versus 75 years; *P*<0.001) and predominantly female (71.7% versus 53%; *P*<0.001). The maintenance of the same mRS score after MT was observed in 100 (23.3%) patients with prestroke mRS score 3 to 4, compared with 2332 (22.1%) patients with mRS score 0 to 2 (*P*=0.556). Mortality was significantly higher in the mRS score 3 to 4 group (n=159 [37.1%] versus n=1939 [18.4%]; *P*<0.001). Successful recanalization (Thrombolysis in Cerebral Infarction score ≥2b) was lower in the mRS score 3 to 4 group (n=333 [71.6%] versus n=8706 [77.7%]; *P*=0.002), while no significant differences in symptomatic intracerebral hemorrhage were found. The benefit of MT was maintained in patients aged 80 to 85 and over 85 years with prestroke mRS score 3 to 4, although mortality remained higher.

**CONCLUSIONS:**

Our data suggest that prestroke disability does not imply less chance of returning to prestroke conditions after MT, even in octogenarians, despite higher mortality and lower recanalization rate. More data are warranted to better understand the benefit of MT in this subgroup of patients.

International guidelines recommend mechanical thrombectomy (MT) for acute ischemic stroke caused by large vessel occlusion in the anterior circulation for patients with a modified Rankin Scale (mRS) score of ≤1.^1,2^ In fact, patients with prestroke disability, defined as an mRS score >1, were initially excluded from clinical trials, resulting in a lack of clinical outcome and safety data for this significant subgroup. Nevertheless, it is estimated that over one-third of patients with acute ischemic stroke due to large vessel occlusion have preexisting disabilities, raising questions about the appropriateness of endovascular treatment for these individuals.^3,4^

Preliminary investigations, mostly single-center studies with limited sample sizes, have suggested potential benefits of MT even in patients with an mRS score >1, though this is accompanied by a significantly increased 90-day mortality rate.^5,6^ The expected benefit quantification, however, depends on the definition of outcomes. The varying inclusion criteria and end points of different studies have complicated the interpretation of results, which remains inconsistent. The standard definition of a favorable outcome, mRS score 0 to 2 at 3 months, is disadvantageous for those with preexisting disabilities (mRS score, 2) and unfeasible for individuals with mRS score of 3 to 4 compared with those with an initial mRS score of 0 to 1.

Moreover, advancements in endovascular techniques, the extension of the treatment window to 24 hours, and broader adoption of these procedures have led to an increasing number of patients undergoing MT. In clinical practice, including patients with an mRS score ≤2 has become routine. Thus, the definition of a favorable outcome requires revision for patients with prestroke disabilities. A recent meta-analysis demonstrated that over a quarter of patients with mRS score 3 to 4 could return to their prestroke condition after MT, indicating potential benefits even for this group.^7^

Although aggregated data suggest a potential benefit of MT in this population, evidence remains limited, particularly for patients with a prestroke mRS score >2, and the scientific community has called for further high-quality data from larger sample sizes.^8,9^ Additionally, there is no information on the impact of age in this cohort undergoing thrombectomy. In this study, we examined the benefit and safety of MT in a population of patients with prestroke mRS score 3 to 4, assessing the likelihood of returning to prestroke conditions compared with those with mRS score 0 to 2, using data from the Italian Registry of Endovascular Treatment in Acute Stroke. We also analyzed the effect of age on outcomes in these patients.

## Methods

### Study Design

The Italian Registry of Endovascular Treatment in Acute Stroke established in 2010, collects clinical and instrumental data on endovascular treatments for acute ischemic stroke. It includes all consecutive patients undergoing these procedures according to current guidelines, with voluntary participation from both academic and hospital institutions. Initially involving 26 centers, the registry has expanded to 55 sites. However, only those with at least 80% data completeness in the main efficacy and safety measures are considered suitable for statistical analysis. For this study, data from 40 centers were used. Further organizational details and the objectives of this multicenter national registry are described elsewhere.^10^ Ethical approval for the Italian Registry of Endovascular Treatment in Acute Stroke was approved by the Ministry of Health in 2015. The use of anonymized data for retrospective studies does not require additional approvals. The study adhered to the STROBE guidelines (Strengthening the Reporting of Observational Studies in Epidemiology) for observational research (Supplemental Material).^11^ Anonymized data can be provided upon reasonable request to the corresponding author.

**Figure 1.**
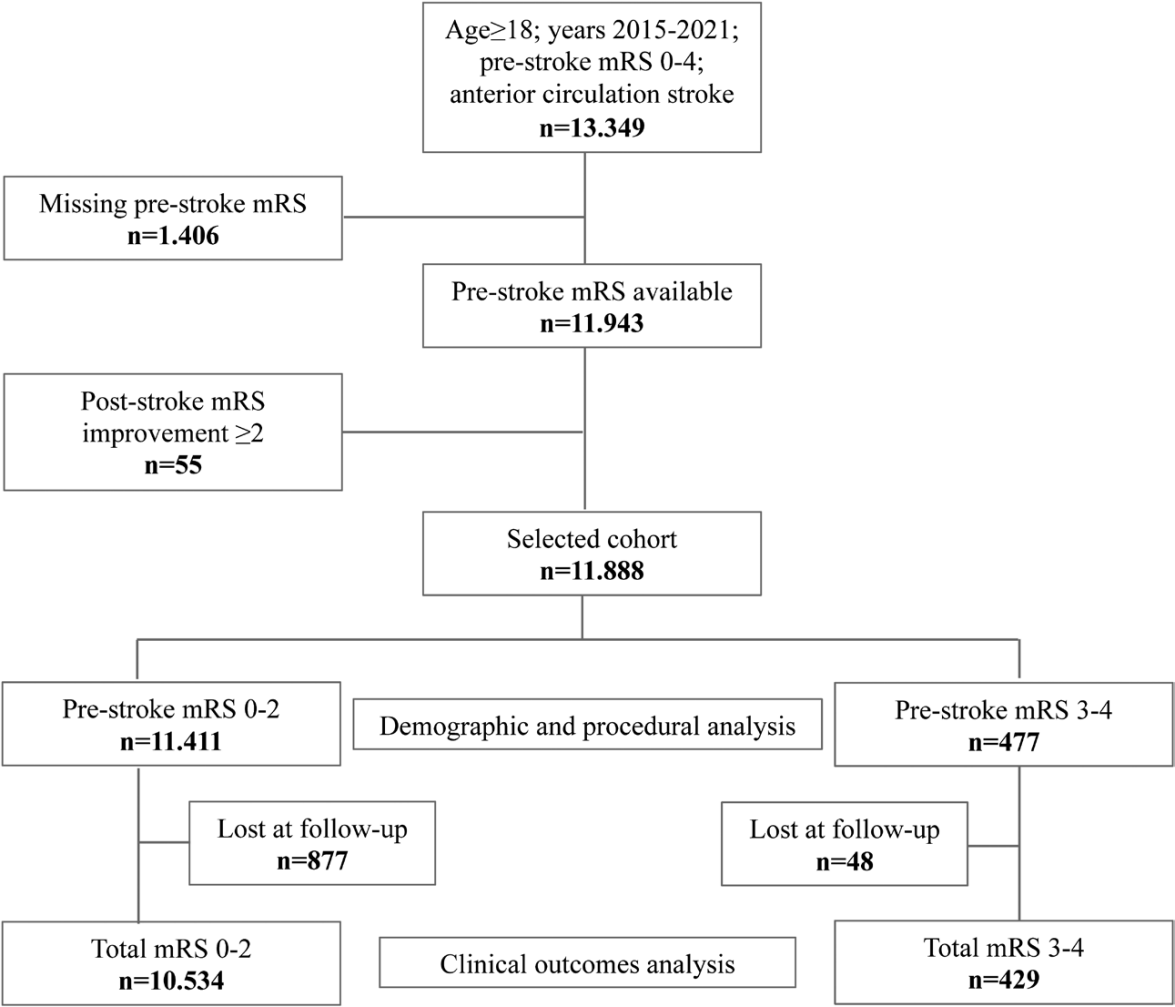
**Patient selection flowchart.** mRS indicates modified Rankin Scale.

The study period spanned from January 2015 to December 2021. We retrospectively included all consecutive patients aged ≥18 years who underwent MT for anterior large vessel occlusion, with or without prior intravenous thrombolysis (IVT). Inclusion criteria for MT aligned with current Italian and international guidelines.^1,12^ Exclusions included posterior circulation strokes, patients lacking a prestroke mRS score, those with a baseline mRS score of 5, and those showing a 90-day mRS improvement of ≥2 points compared with baseline. This exclusion was necessary to account for potential discrepancies in mRS evaluation among operators and transient prestroke disabilities. Instead, an improvement of 1 point was considered as no change from the baseline mRS in the final outcome. The patient selection criteria flowchart is presented in Figure 1.

### Population Characteristics

We collected demographic data (age and sex), clinical information (stroke severity at admission measured by the National Institutes of Health Stroke Scale [NIHSS]), patients’ medical history (vascular risk factors and pre-event medications), radiological features (Alberta Stroke Program Early CT Score, ranging from 0 to 10 where a lower score indicates a larger ischemic area), site of vessel occlusion, recanalization rate measured by the Thrombolysis in Cerebral Infarction scale, and key time intervals for imaging and endovascular procedures.^13,14^

### Outcome Measures

Prestroke and poststroke disability was assessed using the mRS score, defined as follows: 0, no symptoms; 1, no significant disability despite symptoms; 2, slight disability; 3, moderate disability; 4, moderate-to-severe disability; 5, severe disability; and 6, death. Patients were categorized into 2 groups: prestroke mRS score 0 to 2 (absent/minor disability) and mRS score 3 to 4 (moderate/severe disability), with comparisons made between variables. Functional outcomes were measured by the 90-day mRS, obtained through follow-up visits or neurologist-conducted phone calls. Primary outcomes were defined as a good clinical outcome at 90 days (no change in mRS score compared with prestroke) and 90-day mortality (mRS score, 6). Secondary outcomes included the technical success and safety of MT. Safety variables included the rate of symptomatic intracerebral hemorrhage, defined according to the ECASS III criteria as any cerebral hemorrhage accompanied by clinical deterioration, with an increase of ≥4 points on the NIHSS, as well as post-thrombectomy complications such as distal embolization from thrombus fragmentation, inguinal hematoma of any size requiring surgery, and vessel dissection during treatment.^15^ Technical efficacy was assessed by the recanalization rate, based on Thrombolysis in Cerebral Infarction scale values of 2b-3 (successful recanalization with complete filling/perfusion) versus 0-2a (ranging from no perfusion to partial filling). Finally, the effect of age on the primary outcomes was assessed.

### Statistical Analysis

Statistical analysis was performed with the IBM SPSS Statistics (version 29.0) software package. We reported counts and percentages for categorical variables, and median and interquartile range for other variables. For the comparison between groups (mRS score 0–2 versus 3–4), we used 2-tailed Fisher exact test for categorical variables and Mann-Whitney *U* test for other variables. When needed, we estimated effect sizes using adjusted standardized residuals for categorical variables and Hodges-Lehmann median differences for other variables. We used logistic regression analysis to evaluate the relationship between various predictor variables and binary outcome variables reporting *P*, B with SE, and odds ratio (exp(B)) with 95% CIs. As independent variables, we used age, sex, NIHSS, vascular risk factors, successful recanalization, combined IVT+endovascular therapy, and door-to-groin time. For modeling the relationship between age and mRS or mortality, we binned the sample in 20 age-classes, using 5% centiles bins, then we computed the mean of age, mortality, and mRS recovering frequency for every class. We used adjusted R^2^ model selection for choosing the best regression model (among linear, polynomial, or exponential). *P*<0.05 was chosen as the statistical significance threshold.

## Results

### Population Characteristics

For clinical, demographic, and radiological characteristics, 11 411 patients with prestroke mRS score 0 to 2 (96%) were compared with 477 patients with prestroke mRS score 3 to 4 (4%). Of these, 414 (87%) patients presented with prestroke mRS score of 3, while 63 (13%) had a prestroke mRS score of 4. After excluding missing 90-day mRS data, the outcome analysis was conducted between 10 534 (mRS score, 0–2) and 429 (mRS score, 3–4) individuals.

Patients with moderate-to-severe disability were older (82 versus 75 years; *P*<0.001), more frequently female (71.7% versus 53%; *P*<0.001), and had higher presenting NIHSS scores (18 versus 16; *P*<0.001). Additionally, those with prestroke mRS score 3 to 4 exhibited a significantly higher vascular risk profile and greater use of antithrombotic medications (both antiplatelets and anticoagulants), antihypertensives, and statins. No significant differences were noted in radiological parameters, including the Alberta Stroke Program Early CT Score and the site of vascular occlusion, between the groups. Patients with prestroke mRS score 3 to 4 were less likely to receive IVT compared with those with prestroke mRS score 0 to 2 (32% versus 51.1%; *P*<0.001). Regarding MT, there was a significant increase in door-to-imaging and door-to-groin times for patients with greater disability (32 versus 28 and 84 versus 77 minutes, respectively; both *P*<0.001). A nonsignificant trend of shorter procedure duration was observed in patients with prestroke mRS score 3 to 4 (55 versus 60 minutes; *P*=0.058). Detailed clinical, demographic, and radiological analyses with respective significances are provided in Table 1.

**Table 1.**
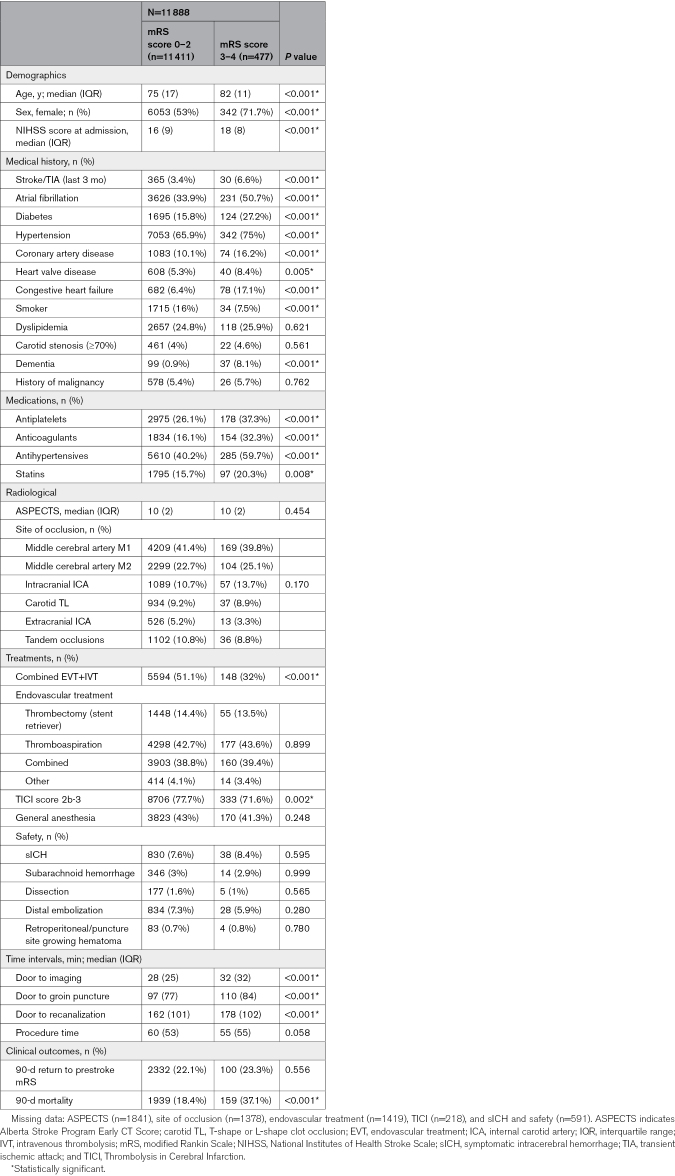
Demographic, Clinical, and Radiological Features, and Outcomes of Patients With Prestroke mRS Scores of 0 to 2 and 3 to 4 Treated With Mechanical Thrombectomy

### Outcomes

**Figure 2.**
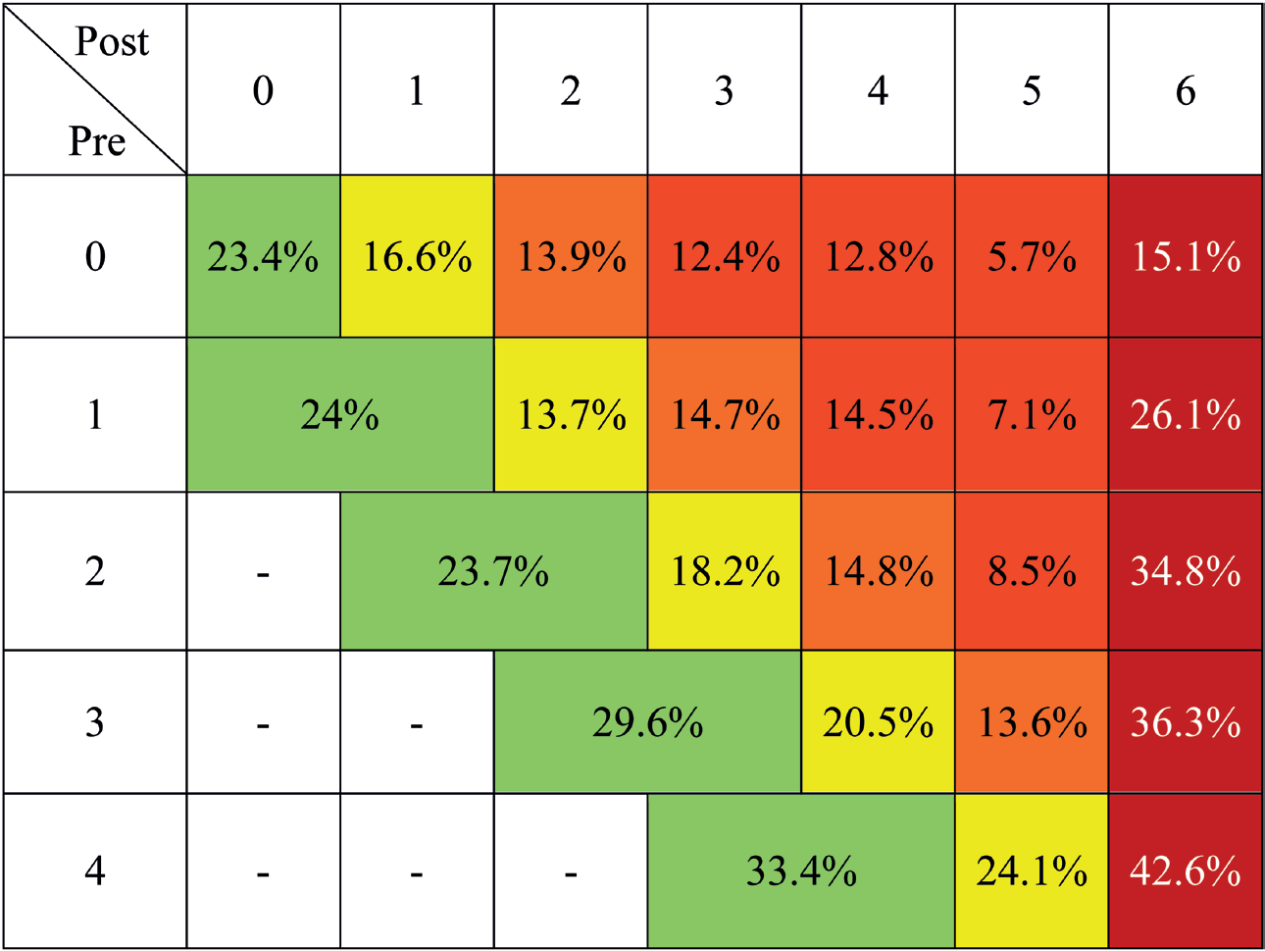
**Changes in modified Rankin Scale (mRS) score and mortality relative to baseline disability (prestroke mRS on the vertical axis, poststroke mRS on the horizontal axis).** Green: no mRS changes compared with baseline disability. Yellow: 1-point worsening shift in mRS. Orange: 2-point worsening shift in mRS. Red: ≥3-point worsening shift in mRS. Dark red: 3-month mortality.

The primary and secondary outcomes are presented in Table 1. The proportion of patients maintaining the same mRS score as their baseline was 23.3% for those with a prestroke mRS score of 3 to 4, compared with 22.1% for patients with a prestroke mRS score of 0 to 2 (*P*=0.556). In contrast, mortality was significantly lower in the group with less prestroke disability (18.4% versus 37.1%; *P*<0.001). After adjusting for age, sex, NIHSS score, vascular risk factors, successful recanalization, IVT+EVT, and door-to-groin time, the odds ratio for mortality was 1.59 for patients with a prestroke mRS score of 3 to 4 (odds ratio, 1.59 [CI, 1.26–2.01]; *P*<0.001). Changes in mRS and mortality relative to baseline disability are detailed in Figure 2.

Regarding secondary outcomes, the rate of successful recanalization (Thrombolysis in Cerebral Infarction score ≥2b) was lower in patients with a prestroke mRS score of 3 to 4 compared with those with mild or no disability (71.6% versus 77.7%; *P*=0.002). MT demonstrated a similar safety profile between groups, with symptomatic intracerebral hemorrhage rates of 8.4% in the mRS score 3 to 4 group versus 7.6% in the mRS score 0 to 2 group (*P*=0.595). Procedural complication rates did not differ significantly between the groups.

Annually, the proportion of patients with a prestroke mRS score of 3 to 4 undergoing thrombectomy averaged 4%, with no significant variation over the 7-year period analyzed (data not shown).

Age stratification revealed that the probability of maintaining the same mRS poststroke as baseline was similar between the 2 groups for patients <80 years of age. Interestingly, this probability increased significantly for patients aged 80 to 85 years and those >85 years of age with a prestroke mRS score of 3 to 4 (Table 2). Conversely, mortality rates remained consistently higher for the prestroke mRS score 3 to 4 group, regardless of age.

**Table 2.**
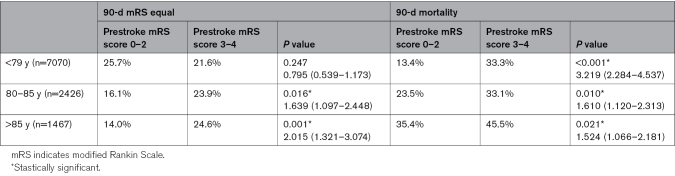
Three-Month Probability of Maintaining the Same mRS Score and Mortality Stratified by Age in Patients With Prestroke mRS Scores of 0 to 2 and 3 to 4

**Figure 3.**
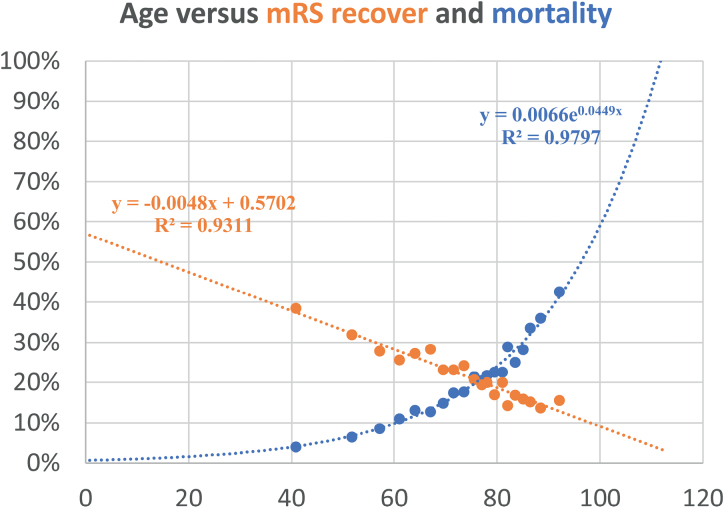
Relationship between age and the risk of death (blue line), and age and the probability of maintaining the same prestroke modified Rankin Scale (mRS) score after stroke treated with mechanical thrombectomy (orange line), independently of baseline mRS.

The intersection of 2 models relating age and the risk of death (blue line), and age and the probability of returning to the same prestroke mRS (orange line), regardless of baseline mRS, is shown in Figure 3.

## Discussion

This study analyzes the largest cohort of patients with preexisting disabilities (mRS score, 3-4) and acute ischemic stroke due to large vessel occlusion who underwent MT. The findings indicate that this subgroup has a similar likelihood of maintaining their baseline disability level compared with those with mild or no prestroke disability. However, the mortality rate is significantly higher in this population. MT was proven to be safe, showing no increased risk of symptomatic intracerebral hemorrhage or procedural complications across the groups analyzed. Notably, we observed a lower recanalization rate in patients with higher baseline disabilities, along with increased times to access imaging and initiate MT in these patients, which correlated with a trend toward shorter duration of the endovascular procedure.

With our criterion for a good outcome established, the favorable clinical outcomes documented are consistent with a recent meta-analysis, showing nearly overlapping return rates to the same initial mRS score (20% versus 23.7% for an mRS score of 2; 27% versus 29.6% for an mRS score of 3; 31% versus 33.6% for an mRS score of 4), while our mortality rates were lower (36% versus 34.8% for an mRS score of 2; 45% versus 36.3% for an mRS score of 3; 61% versus 42.6% for an mRS score of 4).^7^ Interestingly, the clinical benefit appears to be greater in patients with higher prestroke disability, a finding that is consistent with other studies.^16,17^ A possible explanation for this phenomenon is 2-fold: from a clinical perspective, the transition from 0 to 1 or 1 to 2 on the mRS is more nuanced and seemingly easier to achieve compared with the transition from 3 to 4, which more clearly defines a loss of autonomy. Second, patients with mRS score 3 to 4 included in the analysis may represent a hyperselected group by the treating physicians, indicating a selection bias. In fact, a favorable neuroimaging pattern (including perfusion studies) or specific clinical characteristics (such as the type of preexisting disability) may have guided patient selection. These reasons may also explain the observed net benefit in our age-stratified analysis, showing a significant benefit in maintaining the same baseline mRS score in patients with moderate-severe preexisting disability compared with those with mild or no disability as age increases. Taken together, these findings appear particularly notable given the higher burden of comorbidities and increased procedural time intervals observed in the group with prestroke mRS score 3 to 4. Despite these factors, the proportion of patients returning to their premorbid mRS was similar to those with milder or no prestroke disability. This suggests that, although this population presents with additional risk factors, they do not necessarily translate into poorer functional outcomes post-thrombectomy. However, this must be interpreted with caution, as the mechanisms underlying this result remain unclear and may reflect—at least in part—a combination of careful patient selection and effective treatment protocols. This interpretation may also apply to octogenarians, although no conclusive data can be drawn in this regard. Nevertheless, while further studies are needed to confirm our findings and better delineate the predictors of functional recovery in this group, this result holds significant relevance given the aging population and the increasing number of elderly individuals undergoing MT, with potential implications for socioeconomic costs.^4^

The mortality data are also consistent. In agreement with previous studies, mortality in patients with greater prestroke disability is significantly increased at 3 months.^18–20^ Conversely, patients with such disabilities appear to benefit from thrombectomy compared with medical therapy in terms of reduced final mortality, particularly in cases of effective recanalization.^7,21–24^

MT was found to be safe, with nonsignificant rates of procedural complications and symptomatic intracerebral hemorrhage in the analyzed populations. We documented a lower recanalization rate in the mRS score 3 to 4 group, aligning with prior observations by Florent et al^20^ but contrasting with other studies that reported no differences in recanalization rates.^5,9,19,21^ This finding was associated with increased times to access imaging and start treatment. We speculate that the longer door-to-imaging and door-to-treatment times may be partially due to the need for more extensive anamnesis on prestroke conditions during patient selection. Physicians likely required additional time to assess the overall prognosis, comorbid conditions, and potential benefits of MT in these patients, which contributed to the delays. Moreover, obtaining a thorough medical history and consulting family members or caregivers for patients with preexisting disabilities may have also played a role in extending these time intervals. Similarly, we observed a trend toward shorter treatment duration in the angiography suite, possibly due to fewer thrombectomy passes being performed in patients with preexisting disabilities, which consequently reduces recanalization rates. Certainly, we cannot exclude that other factors not systematically available from the registry data (such as more complex vascular anatomy or a greater clot burden) may have contributed to this result. Nevertheless, favorable outcome results were comparable between groups, suggesting that the early termination of MT in case of failure should not be justified in this population and also highlighting the importance of optimizing workflow efficiency in this subgroup.

Our study has several limitations. The first is the retrospective design. As registry data, outcome measures may vary between centers due to the absence of centralized adjudication. Additionally, we lack a control group for comparison. Furthermore, patients with preexisting disability undergoing treatment may represent a highly selected group, excluding those deemed too frail or at higher risk of complications, while including those with favorable advanced neuroimaging features. However, with the available registry data, it was not possible to identify patients who may have been selected based on perfusion imaging. This selection bias could result in an overestimation of good outcomes in the group with greater preexisting disability. Moreover, we do not have details on which kind of disability, whether neurological or orthopedic, was responsible for the prestroke mRS. We also lack data concerning the type of rehabilitation between discharge and 90-day follow-up and differences in the quality of rehabilitation in different regions of Italy. Finally, we cannot rule out interference in outcomes, including bleeding complications, due to the different proportions of patients undergoing IVT in the analyzed groups.

We also clarify that, although the principles of patient selection, procedural safety, and the role of MT in prestroke disability populations likely extend beyond regional differences, the results of this study pertain to a specific national health care system with distinct access to acute stroke treatment and poststroke rehabilitation pathways. Therefore, the generalizability of the results and their applicability to other health care systems must be further investigated.

Despite all limitations, these real-world data suggest the unavoidable need to pay careful selection of patients with relevant prestroke disability based on a detailed medical history and neuroimaging predictors. Our data suggest the efficacy and safety of MT in selected patients with moderate-to-severe prestroke disability (mRS score, 3–4) and also in association with advanced age. Although careful patient selection is mandatory, prestroke disability alone does not seem to be a sufficient criterion to exclude patients from endovascular treatment. Randomized controlled trials are needed to assess the outcomes and safety of MT in patients with prestroke disability, providing insights to refine clinical practice and patient selection. We encourage future research to address this important gap and guide decision-making in this complex population.

## Article Information

### Sources of Funding

None.

### Disclosures

Dr Nappini: consultancy for Medtronic, Cerenovus, Stryker, and Balt. Dr Cappellari: consultancy or advisory board fees or speaker honoraria from Pfizer/Bristol Myer Squibb and Daiichi Sankyo. Dr Romano: consultant at Balt Italy, Balt Europe, Penumbra, Microvention, Stryker Neuro, Wallaby-Phenox. Dr Sacco: compensation from Pfizer Canada, Inc, Lundbeck, Eli Lilly and Company, Allergan, Boehringer Ingelheim, AstraZeneca, Novo Nordisk, Abbott Canada, Teva Pharmaceutical Industries, and Novartis for consultant services; compensation from Novartis for other services; employment by Università degli Studi dell’Aquila; intellectual: president-elect of the European Stroke Organisation, second vice president of the European Headache Federation, specialty chief editor in Headache and Neurogenic Pain for *Frontiers in Neurology*, associate editor for *The Journal of Headache and Pain*, and assistant editor for *Stroke*. Dr Cavasin: compensation from MicroVention, Inc, and from Stryker for consultant services. Dr Limbucci: compensation from Balt, Johnson & Johnson Health Care Systems, Inc, Medtronic, Inc, Stryker Corporation, MicroVention, Inc, and Penumbra, Inc, for consultant services. The other authors report no conflicts.

### Supplemental Material

STROBE Checklist

## References

[1] Powers WJ Rabinstein AA Ackerson T Adeoye OM Bambakidis NC Becker K Biller J Brown M Demaerschalk BM Hoh B. Guidelines for the early management of patients with acute ischemic stroke: 2019 update to the 2018 guidelines for the early management of acute ischemic stroke: a guideline for healthcare professionals from the American Heart Association/American Stroke Association. Stroke. 2019;50:e344–e418. doi: 10.1161/STR.0000000000000211 31662037 10.1161/STR.0000000000000211

[2] Turc G Bhogal P Fischer U Khatri P Lobotesis K Mazighi M Schellinger PD Toni D de Vries J White P. European Stroke Organisation (ESO) - European Society for Minimally Invasive Neurological Therapy (ESMINT) guidelines on mechanical thrombectomy in acute ischemic Stroke. J Neurointerv Surg. 2023;15:e8. doi: 10.1136/neurintsurg-2018-014569 30808653 10.1136/neurintsurg-2018-014569

[3] Gil-Salcedo A Dugravot A Fayosse A Landré B Jacob L Bloomberg M Sabia S Schnitzler A. Pre-stroke disability and long-term functional limitations in stroke survivors: findings from more of 12 years of follow-up across three international surveys of aging. Front Neurol. 2022;13:888119. doi: 10.3389/fneur.2022.888119 35775052 10.3389/fneur.2022.888119 PMC9237334

[4] Ganesh A Luengo-Fernandez R Pendlebury ST Rothwell PM. Long-term consequences of worsened poststroke status in patients with premorbid disability. Stroke. 2018;49:2430–2436. doi: 10.1161/STROKEAHA.118.022416 30355105 10.1161/STROKEAHA.118.022416 PMC6159688

[5] Goldhoorn RB Verhagen M Dippel DWJ van der Lugt A Lingsma HF Roos YBWEM Majoie CBLM Vos JA Boiten J van Zwam WH; MR CLEAN Registry Investigators. Safety and outcome of endovascular treatment in prestroke-dependent patients. Stroke. 2018;49:2406–2414. doi: 10.1161/STROKEAHA.118.022352 30355090 10.1161/STROKEAHA.118.022352

[6] Oesch L Arnold M Bernasconi C Kaesmacher J Fischer U Mosimann PJ Jung S Meinel T Goeldlin M Heldner M. Impact of pre-stroke dependency on outcome after endovascular therapy in acute ischemic stroke. J Neurol. 2021;268:541–548. doi: 10.1007/s00415-020-10172-3 32865630 10.1007/s00415-020-10172-3 PMC7880932

[7] Yang JC Bao QJ Guo Y Chen SJ Zhang JT Zhang Q Zhou P Yang MF. Endovascular thrombectomy in acute ischemic stroke patients with prestroke disability (mRS ≥2): a systematic review and meta-analysis. Front Neurol. 2022;13:971399. doi: 10.3389/fneur.2022.971399 36188370 10.3389/fneur.2022.971399 PMC9520304

[8] Wechsler PM Leslie-Mazwi T Mistry EA. Endovascular thrombectomy in patients with preexisting disability: a review. Stroke Vasc Interv Neurol. 2024;4:e001326. doi: 10.1161/SVIN.124.001326 10.1161/SVIN.124.001326 PMC12778517 41584483

[9] Larsson A Karlsson C Rentzos A Schumacher M Abrahamson M Allardt A Brederlau A Ceder E Davidson M Dunker D. Do patients with large vessel occlusion ischemic stroke harboring prestroke disability benefit from thrombectomy? J Neurol. 2020;267:2667–2674. doi: 10.1007/s00415-020-09882-5 32410019 10.1007/s00415-020-09882-5 PMC7419353

[10] Mangiafico S Pracucci G Saia V Nencini P Inzitari D Nappini S Vallone S Zini A Fuschi M Cerone D. The Italian registry of endovascular treatment in acute stroke: rationale, design and baseline features of patients. Neurol Sci. 2015;36:985–993. doi: 10.1007/s10072-014-2053-5 25567080 10.1007/s10072-014-2053-5

[11] von EE Altman DG Egger M Pocock SJ Gøtzsche PC Vandenbroucke JP; STROBE Initiative. The Strengthening the Reporting of Observational Studies in Epidemiology (STROBE) statement: guidelines for reporting observational studies. Int J Surg. 2014;12:1495–1499. doi: 10.1016/j.ijsu.2014.07.013 25046131 10.1016/j.ijsu.2014.07.013

[12] SPREAD (Stroke Prevention and Educational Awareness Diffusion). Italian Guidelines for Stroke Prevention and Management. 8th ed. Accessed August 20, 2024. https://isa-aii.com 10.1007/s100720070112 10938196

[13] Barber PA Demchuk AM Zhang J Buchan AM. Validity and reliability of a quantitative computed tomography score in predicting outcome of hyperacute stroke before thrombolytic therapy. ASPECTS Study Group. Alberta Stroke Programme Early CT Score. Lancet. 2000;355:1670–1674. doi: 10.1016/s0140-6736(00)02237-6 10905241 10.1016/s0140-6736(00)02237-6

[14] Higashida RT Furlan AJ Roberts H Tomsick T Connors B Barr J Dillon W Warach S Broderick J Tilley B; Technology Assessment Committee of the American Society of Interventional and Therapeutic Neuroradiology. Trial design and reporting standards for intra-arterial cerebral thrombolysis for acute ischemic stroke. Stroke. 2003;34:e109–e137. doi: 10.1161/01.STR.0000082721.62796.09 12869717 10.1161/01.STR.0000082721.62796.09

[15] Hacke W Kaste M Bluhmki E Brozman M Dávalos A Guidetti D Larrue V Lees KR Medeghri Z Machnig T; ECASS Investigators. Thrombolysis with alteplase 3 to 4.5 hours after acute ischemic stroke. N Engl J Med. 2008;359:1317–1329. doi: 10.1056/NEJMoa0804656 18815396 10.1056/NEJMoa0804656

[16] Regenhardt RW Young MJ Etherton MR Das AS Stapleton CJ Patel AB Lev MH Hirsch JA Rost NS Leslie-Mazwi TM. Toward a more inclusive paradigm: thrombectomy for stroke patients with pre-existing disabilities. J Neurointerv Surg. 2021;13:865–868. doi: 10.1136/neurintsurg-2020-016783 33127734 10.1136/neurintsurg-2020-016783 PMC8365380

[17] Seker F Pfaff J Schönenberger S Herweh C Nagel S Ringleb PA Bendszus M Möhlenbruch MA. Clinical outcome after thrombectomy in patients with stroke with premorbid modified Rankin Scale scores of 3 and 4: a cohort study with 136 patients. AJNR Am J Neuroradiol. 2019;40:283–286. doi: 10.3174/ajnr.A5920 30573460 10.3174/ajnr.A5920 PMC7028628

[18] Slawski DE Salahuddin H Shawver J Kenmuir CL Tietjen GE Korsnack A Zaidi SF Jumaa MA. Mechanical thrombectomy in elderly stroke patients with mild-to-moderate baseline disability. Interv Neurol. 2018;7:246–255. doi: 10.1159/000487333 29765394 10.1159/000487333 PMC5939652

[19] Salwi S Cutting S Salgado AD Espaillat K Fusco MR Froehler MT Chitale RV Kirshner H Schrag M Jasne A. Mechanical thrombectomy in patients with ischemic stroke with prestroke disability. Stroke. 2020;51:1539–1545. doi: 10.1161/STROKEAHA.119.028246 32268851 10.1161/STROKEAHA.119.028246 PMC7367056

[20] Florent EG Barbara C Marc F Maeva K Fouzi B Lucie DS Charlotte C Nicolas B Hilde H. Clinical outcomes and safety of mechanical thrombectomy for acute ischaemic stroke in patients with pre-existing dependency. J Stroke Cerebrovasc Dis. 2021;30:105848. doi: 10.1016/j.jstrokecerebrovasdis.2021.105848 33991770 10.1016/j.jstrokecerebrovasdis.2021.105848

[21] McDonough RV Ospel JM Majoie CBLM Saver JL White P Dippel DWJ Brown SB Demchuk AM Jovin TG Mitchell PJ; HERMES Collaborators. Clinical outcome of patients with mild pre-stroke morbidity following endovascular treatment: a HERMES substudy. J Neurointerv Surg. 2023;15:214–220. doi: 10.1136/neurintsurg-2021-018428 35210331 10.1136/neurintsurg-2021-018428

[22] Sprügel MI Sembill JA Kremer S Gerner ST Knott M Hock S Engelhorn T Dörfler A Huttner HB Schwab S. Evaluation of functional recovery following thrombectomy in patients with large vessel occlusion and prestroke disability. JAMA Netw Open. 2022;5:e2227139. doi: 10.1001/jamanetworkopen.2022.27139 35972737 10.1001/jamanetworkopen.2022.27139 PMC9382438

[23] Tanaka K Yamagami H Yoshimoto T Uchida K Morimoto T Toyoda K Sakai N Yoshimura S. Endovascular therapy for acute ischemic stroke in patients with prestroke disability. J Am Heart Assoc. 2021;10:e020783. doi: 10.1161/JAHA.121.020783 34284599 10.1161/JAHA.121.020783 PMC8475666

[24] Kastrup A Roth C Politi M Alexandrou M Hildebrandt H Schröter A Papanagiotou P. Endovascular therapy vs. thrombolysis in pre-stroke dependent patients with large vessel occlusions within the anterior circulation. Front Neurol. 2021;12:666596. doi: 10.3389/fneur.2021.666596 34149598 10.3389/fneur.2021.666596 PMC8206778

